# Lymphangiome kystique orbitaire envahissant le globe oculaire: à propos d'un cas

**DOI:** 10.11604/pamj.2015.21.237.7490

**Published:** 2015-07-31

**Authors:** Wafa Ammari, Olfa Berriche

**Affiliations:** 1 Service d'Ophtalmologie, Hôpital Taher Sfar, Mahdia, Tunisie; 2 Service de Médecine Interne, Hôpital Taher Sfar, Mahdia, Tunisie

**Keywords:** Lymphangiome, oeil, tumorectomie, Lymphangioma, eye, lumpectomy

## Image en medicine

Le lymphangiome orbitaire est une tumeur bénigne à développement progressif, qui se présente sous forme de vaisseaux à parois fines et à lumières dilatées complètement isolées de la circulation artérioveineuse. Le lymphangiome peut se localiser au niveau de la conjonctive, des paupières ou de l'orbite. A ce propos, nous rapportons un cas de lymphangiome orbitaire envahissant le globe oculaire et dont la présentation initiale mimait celle d'un rétinoblastome. Patiente âgée de 19 ans, se plaignant d'une exophtalmie droite et d'une baisse de la vision de l’œil droit évoluant depuis 4 ans. L'examen des annexes objectivait une exophtalmie majeure compliquée d'une ankylose du globe oculaire droit. La photographie du fond d’œil droit montrait la présence d'une masse rétinienne blanchâtre, associée à un essaimage vitréen fait de formations floconneuses. L’échographie oculaire en mode brillance montrait un épaississement hyperéchogène de la rétine. L'IRM orbito-cérébrale montrait une lésion kystique intra orbitaire en rapport avec un lymphangiome. Devant l’évolution longue de la maladie et l'aspect de la masse à l'imagerie, le diagnostic de rétinoblastome a été écarté. La patiente a subit une énucléation et une tumorectomie. L’étude histopathologique de la pièce opératoire a révélé un lymphangiome.

**Figure 1:**
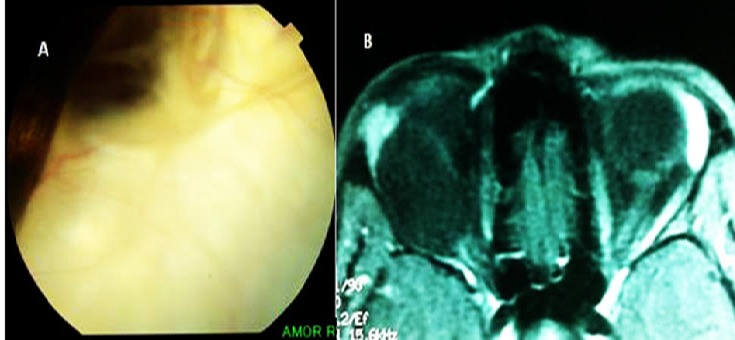
A) photographie du fond d’œil droit: présence d'une masse rétinienne du pôle postérieur, blanchâtre, associée à un essaimage vitréen fait de formations floconneuses d'aspect spéculé; B) IRM orbito-cérébrale: coupe axiale pondérée T1 après injection de gadolinium: masse en hyposignal qui comprime le nerf optique et refoule le muscle droit latéral

